# Superior Ophthalmic Vein Embolism Following Forehead Augmentation with Autologous Fat Injection

**DOI:** 10.1007/s00266-021-02414-0

**Published:** 2021-07-06

**Authors:** Bunyada Putthirangsiwong, Varan Vongsilpavattana, Sasikant Leelawongs, Ekachat Chanthanaphak, Padcha Tunlayadechanont, Weerawan Chokthaweesak

**Affiliations:** 1 Department of Ophthalmology, Faculty of Medicine Ramathibodi Hospital, Mahidol University, 270 Rama VI Road, Ratchathewi, Bangkok, 10400 Thailand; 2 Department of Interventional Neuroradiology, Ramathibodi Hospital, Mahidol University, Bangkok, Thailand; 3 Department of Radiology, Ramathibodi Hospital, Mahidol University, Bangkok, Thailand

**Keywords:** Autologous fat injection, Fat embolism, Superior ophthalmic vein, Vein occlusion

## Abstract

**Background:**

Facial rejuvenation and reconstruction with autologous fat injection are a common and effective procedure used worldwide. Most surgeons and patients are satisfied with the favorable outcomes. However, catastrophic complications from arterial and venous occlusion resulting in visual loss and stroke may occur.

**Case presentation:**

We herein report a case of isolated venous occlusion from fat embolism. The patient developed acute painful proptosis and blurred vision of her right eye while undergoing an esthetic autologous fat injection into her forehead. Based on her clinical manifestations and radiologic findings, the patient was diagnosed with superior ophthalmic vein occlusion. Symptomatic and supportive treatments were given. Spontaneous clinical improvement occurred without secondary complications. Therefore, the initially planned endovascular therapy with transfemoral transvenous embolectomy of the right superior ophthalmic vein was canceled.

**Conclusions:**

Facial augmentation with autologous fat injection can cause superior ophthalmic vein embolism. Surgeons should therefore perform this procedure very cautiously. Prompt ophthalmological evaluation and proper management are important for improving clinical outcomes.

**Level of Evidence V:**

This journal requires that authors assign a level of evidence to each article. For a full description of these Evidence-Based Medicine ratings, please refer to the Table of Contents or the online Instructions to Authors www.springer.com/00266.

## Introduction

Autologous fat injection for facial rejuvenation and reconstruction is a common and effective procedure that is being increasingly performed worldwide. The outcome is favorable in most cases as reflected by the high rate of patient and surgeon satisfaction [1]. However, some patients develop devastating and irreversible vascular complications such as visual loss from ophthalmic or central retinal arterial occlusion or stroke from middle cerebral arterial occlusion [2–11]. Other complications include asymmetry, skin irregularity, prolonged edema, graft hypertrophy, fat necrosis, infection, erythema, telangiectasia, activation of acne, granuloma formation, soft tissue necrosis due to vascular obstruction, and anaphylaxis [12–14].

Venous embolism after facial injection of fillers has been rarely reported in the literature. We herein describe a case of superior ophthalmic vein embolism following autologous fat injection into the forehead area.

### Case Report

A 32-year-old healthy woman presented to our hospital with sudden onset of painful proptosis and decreased vision in her right eye. These symptoms had appeared 30 minutes prior to arrival. She had undergone cosmetic forehead augmentation with autologous fat injection at another clinic. An approximately 10-mL fat graft was harvested from her right medial thigh and injected into her right forehead under local anesthesia. During the procedure, she developed painful bulging of her right eye with blurred vision. The injection was terminated and the patient was immediately referred to our hospital. She denied a history of surgery or trauma.

**Fig. 1:**
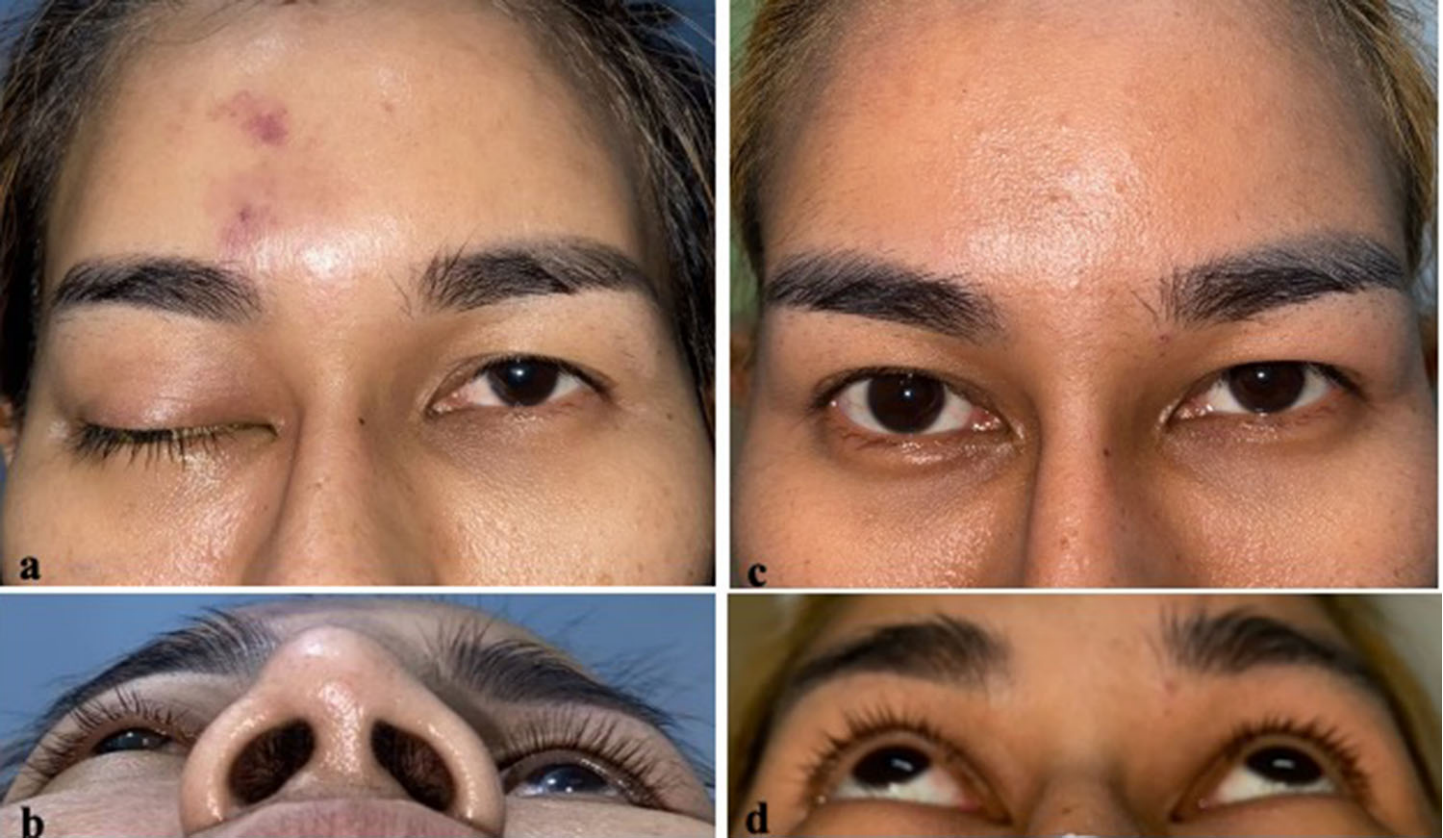
**a**, **b** Photographs obtained before treatment, showing proptosis and droopiness of the patient’s right eye. **c**, **d** Photographs obtained 1 month after treatment, showing clinical improvement

Upon arrival at our hospital, the patient was fully conscious and afebrile. She had no tachypnea, dyspnea, or petechiae on her skin. Physical examination revealed needle marks with bruits on the right side of her forehead. Her vital signs were stable. Ophthalmologic examination demonstrated visual acuity of 20/40 with a positive relative afferent pupillary defect in her right eye. The patient exhibited right eye bulging and droopiness of her right upper eyelid (Fig. 1a). The intraocular pressure of her right eye was 44 mmHg. Anterior segment and fundus examination findings were within normal limits. Extraocular movements were limited in all directions. Decreased sensation was present in the right forehead and right upper eyelid regions. Ophthalmic examination of the left eye was unremarkable. Her muscle strength was grade 5 in all extremities. Initial management involved immediate administration of oral acetazolamide at 500 mg followed by 250 mg every 6 hours, 0.5% timolol eye drops every 12 hours, 0.15% brimonidine-P eye drops every 12 hours, and intravenous amoxicillin/clavulanic acid at 1.2 g every 8 hours. Her intraocular pressure gradually improved after beginning these medical treatments. Therefore, further canthotomy and cantholysis were postponed.

**Fig. 2:**
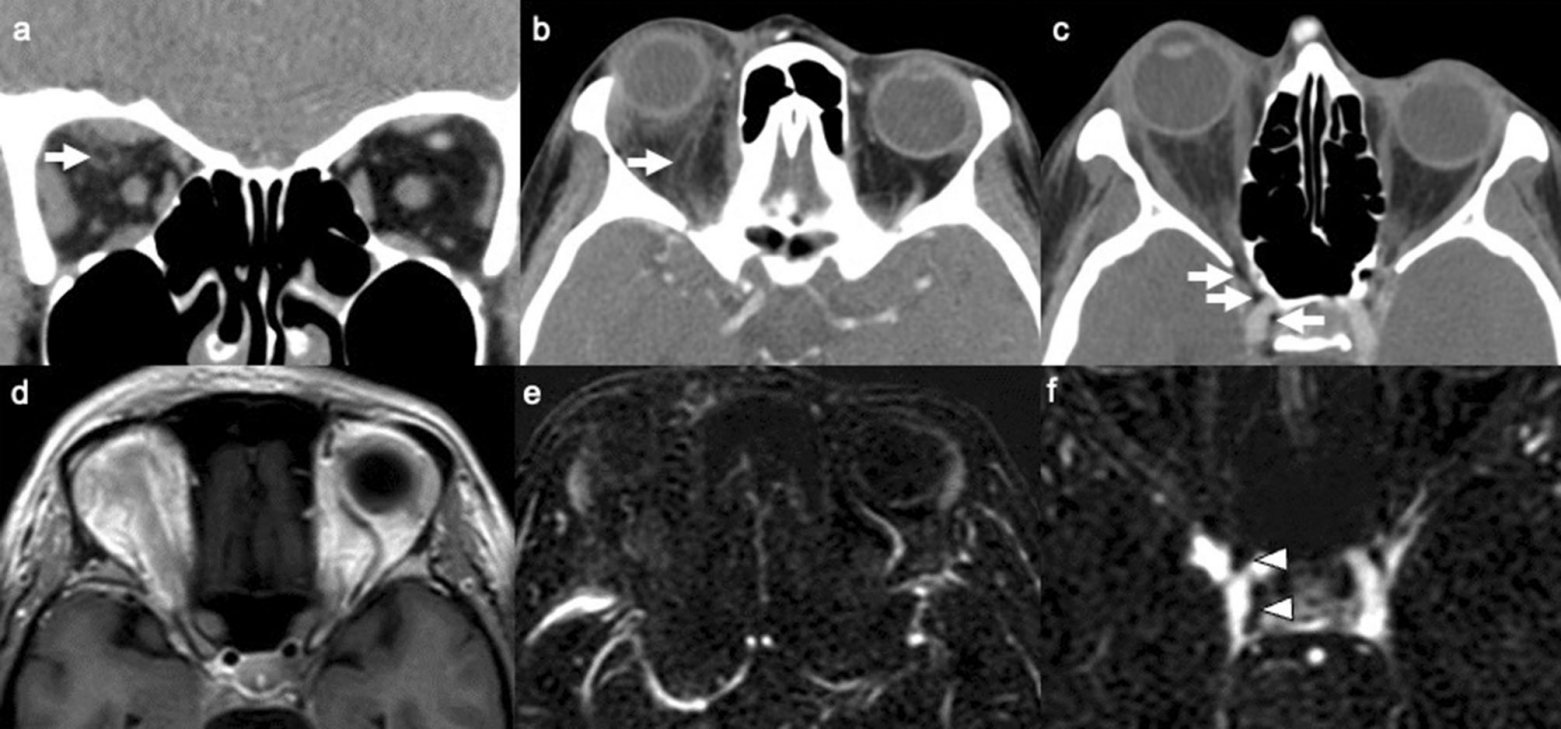
**a**–**c** Contrast-enhanced orbit computed tomography showed fat attenuation filling defects in the dilated right superior ophthalmic vein and right cavernous sinus (white arrow). Right proptosis, right retrobulbar fat reticulation, and right extraocular muscle enlargement from orbital congestion were demonstrated. **d** On T1-weighted images, loss of normal flow void with abnormally increased signal intensity of fat embolism was seen in the right superior ophthalmic vein. **e**, **f** Magnetic resonance venography demonstrated no contrast filling in the right superior ophthalmic vein and few small filling defects in the right cavernous sinus (arrowhead)

Emergency contrast-enhanced orbit computed tomography revealed intraluminal hypoattenuation filling defects, which were suspected to be fat attenuation (−60 HU), in the dilated right superior ophthalmic vein and right cavernous sinus. Right eye proptosis, right retrobulbar fat reticulation, and enlargement of the right extraocular muscles were also demonstrated (Fig. 2a–c). The brain parenchyma was normal without acute infarction, acute hemorrhage, or abnormal enhancing lesions. T1-weighted and T2-weighted magnetic resonance imaging (MRI) showed a hyperintense lesion, which was similar in intensity to the adjacent orbital and subcutaneous fat, in the right superior ophthalmic vein with loss of its normal flow void (Fig. 2d). Postcontrast fat-saturated T1-weighted MRI demonstrated complete suppression of fat signal intensity within the lesion. Magnetic resonance venography revealed filling defects in the right superior ophthalmic vein and right cavernous sinuses (Fig. 2e, f). The bilateral optic nerves exhibited normal signal intensity.

The diagnosis of superior ophthalmic vein occlusion was made based on the patient’s clinical examination and radiologic findings. After a multidisciplinary conference, vascular recanalization via a transfemoral transvenous embolectomy of the right superior ophthalmic vein was scheduled. 14 hours after hospitalization while the procedure was being prepared, the patient’s symptoms spontaneously improved. She felt less pain in her right eye, and the swollen eyelid and proptosis slightly decreased. Her visual acuity recovered to 20/20 with a negative relative afferent pupillary defect in her right eye. Extraocular movements were unrestricted in all directions with the exception of only a 20% limitation in the right upward gaze. Because her condition gradually resolved, plans for endovascular treatment were abandoned.

Despite her clinical improvement, the patient was additionally given intravenous methylprednisolone at 1000 mg and was thereafter treated with oral prednisolone at 1 mg/kg/day for 2 weeks. She was discharged without serious sequelae after 5 days of hospitalization.

**Fig. 3:**
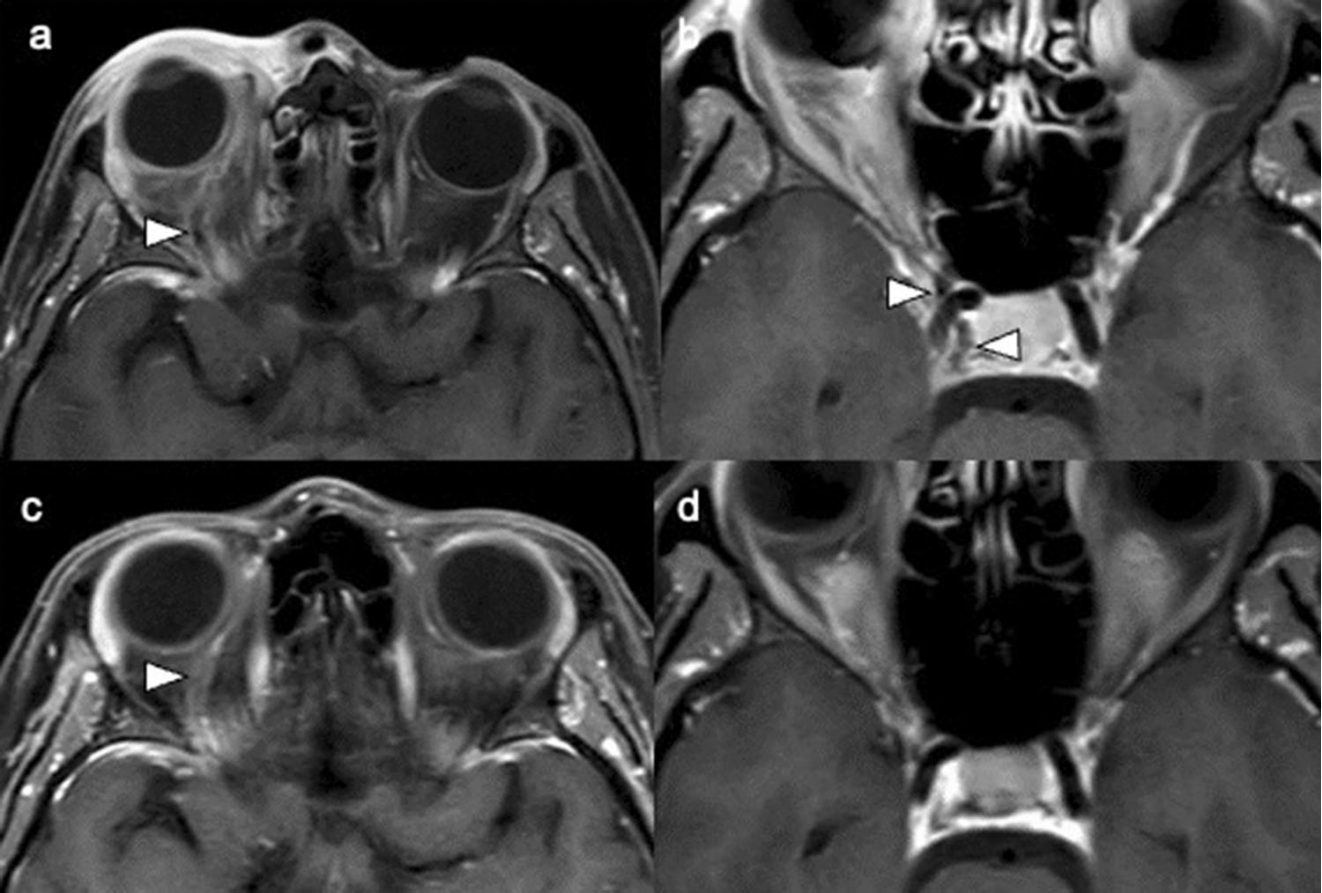
Contrast-enhanced fat-saturated T1-weighted magnetic resonance imaging of the orbit showed **a**, **b** filling defects in the dilated right superior ophthalmic vein and cavernous sinus (arrowhead). **c**, **d** The filling defects had partially resolved on follow-up imaging

At the 1-month follow-up, the patient had made a complete clinical recovery (Fig. 1b). Repeat MRI showed a decrease in the amount of filling defects in the right superior ophthalmic vein and right cavernous sinus with a significant reduction of right orbital congestion (Fig. 3).

## Discussion

Facial fat grafting was first introduced for treatment of scar contracture by Gustav Neuber in 1893 [15]. Because autologous fat is biocompatible, relatively viable when integrated into the surrounding tissue in the surgical site, and easily accessible in most patients, it has become an ideal and commonly used soft tissue filler for reconstructive and cosmetic indications. Several techniques in fat harvesting, fat preparation, and fat grafting pioneered by Coleman have been evolving during the past few decades [13, 16]. Autologous fat injection has been increasingly used since the development of liposuction and is currently popular worldwide. According to pre-existing data, autologous fat grafting is effective and safe for the treatment of facial contour deformities with a low rate of complications [1, 13]. Complications of autologous fat grafting include blindness and stroke from vascular embolism, asymmetry, skin irregularities, prolonged edema, graft hypertrophy, fat necrosis, infection, erythema, telangiectasia, activation of acne, granuloma formation, soft tissue necrosis due to localized vascular obstruction, and anaphylaxis [12–14]. A rare but catastrophic complication is fat embolism, which is a result of migration of the fat graft into the vasculature. Several reports have described patients who developed internal and external carotid artery embolism after facial autologous fat injection, resulting in visual loss and/or cerebral infarction [3–9, 11, 17–19]. Lee et al. [20] reported concurrent central retinal artery occlusion with ophthalmic vein thrombosis after subcutaneous paraffin injection into the forehead. However, there is no documented evidence of an isolated venous embolism/thrombosis following facial fat injection in the literature.

The superior ophthalmic vein is the major draining venous system of the orbit. This vein originates in the superonasal orbit and runs with the superior orbital artery to drain into the cavernous sinus. Superior ophthalmic vein occlusion is an extremely rare condition, and its diagnosis is based on clinical manifestations and radiologic findings. The clinical presentation includes painful proptosis, orbital swelling, eyelid swelling, chemosis, limited extraocular motility, and impaired visual function. Postcontrast computed tomography or MRI demonstrates dilatation of the superior ophthalmic vein with internal filling defects, soft tissue stranding around the superior ophthalmic vein and within the orbital fat, and enlargement of the extraocular muscles [21]. The etiologies can be classified into septic or aseptic causes. Septic causes of superior ophthalmic vein occlusion involve infection of the orbit, paranasal sinus, teeth, and face. Aseptic causes of superior ophthalmic vein occlusion can be a result of altered blood flow from anatomical or systemic conditions such as vascular malformations, facial trauma, autoimmune diseases (e.g., thyroid eye disease, systemic lupus erythematosus, or ulcerative colitis), hematologic disorders (e.g., hereditary hemorrhagic telangiectasia, antiphospholipid syndrome, or sickle cell trait), hormone therapies (e.g., tamoxifen or oral contraceptive pills), neoplasms, Tolosa–Hunt syndrome, and idiopathic orbital inflammatory disease [22]. To the best of our knowledge, this is the first case report of superior ophthalmic vein occlusion from fat embolism following facial autologous fat injection.

The pathogenesis of fat embolism after facial autologous fat injection may be related to the abundant connections among facial vascular networks. There are arterial communications between internal and external carotid artery through facial arteries and a venous drainage system between facial veins, superior ophthalmic vein and cavernous sinus. In our case, we postulate that the fat particles were directly injected into the superior orbital vein and reached the superior ophthalmic vein through the normal venous flow. Additionally, these fat particles were likely pushed retrogradely into the superior ophthalmic vein by the pressure of the injection. This hypothesis is supported by the time of onset (i.e., immediately after the procedure). Theoretically, intravasated fat fragments can travel through the pulmonary circulation, causing respiratory insufficiency, and gain access to the systemic circulation, resulting in alteration of consciousness, renal failure, retinal arterial occlusion, myocardial ischemia, and petechial rash (fat embolism syndrome) [23, 24].

The effectiveness of various treatments of fat embolism remains controversial. The mainstay of treatment is early recognition and supportive therapy. Corticosteroids have been suggested as prophylactic agents that reduce the incidence and severity of fat embolism syndrome [24]. A few authors have recommended corticosteroids as anti-inflammatory agents through their stabilization of membranes, control of free fatty acid levels, and prevention of complement-mediated leukocyte aggregation [25]. Moreover, vascular recanalization can be achieved by prompt utilization of mechanical lipectomy devices [19, 26]. Our patient responded well to symptomatic and supportive treatment. She fortunately recovered spontaneously without developing secondary fat embolism syndrome. Although an invasive mechanical lipectomy procedure was initially planned, it was ultimately not needed. In this case, a systemic corticosteroid regimen was given for the purpose of inflammatory reduction and prevention of probable fat embolism syndrome. This case reminds us that facial fat injection may cause this rare but potentially disastrous complication. Therefore, preventive strategies are of utmost importance. Surgeons should pay particular attention to the anatomy of both the arterial networks and venous systems around the orbit. Aspiration before injection should be done to ensure that the needle is not entering the vessels. Furthermore, fat grafts should be injected in a slow and gentle manner while performing the procedure [12, 27, 28].

## Conclusion

Iatrogenic intravasation of fat grafts into the ocular venous system after facial autologous fat injection can cause superior ophthalmic vein embolism, resulting in acute painful proptosis, diplopia, and vision loss. To avoid these devastating complications, surgeons should perform this procedure with particular caution. Prompt ophthalmologic evaluation and proper management are absolutely vital for improving clinical outcomes.

## References

[1] Krastev TK, Beugels J, Hommes J, Piatkowski A, Mathijssen I, van der Hulst R (2018). Efficacy and safety of autologous fat transfer in facial reconstructive surgery: a systematic review and meta-analysis. JAMA Fac Plast Surg.

[2] Ansari ZA, Choi CJ, Rong AJ, Erickson BP, Tse DT (2019). Ocular and cerebral infarction from periocular filler injection. Orbit.

[3] Lazzeri S, Figus M, Nardi M, Lazzeri D, Agostini T, Zhang YX (2013). Iatrogenic retinal artery occlusion caused by cosmetic facial filler injections. Am J Ophthalmol.

[4] Liu H, Chen D, Zhang J (2020). Ophthalmic artery occlusion after forehead autologous fat injection. Retin Cases Brief Rep.

[5] Liu H, Wu X, Zhang X, Niu C, Zhu H (2019). Internal carotid artery embolism after autologous fat injection for temporal augmentation. Aesthet Plast Surg.

[6] Park KH, Kim YK, Woo SJ, Kang SW, Lee WK, Choi KS, Kwak HW, Yoon IH, Huh K, Kim JW (2014). Iatrogenic occlusion of the ophthalmic artery after cosmetic facial filler injections: a national survey by the Korean retina society. JAMA Ophthalmol.

[7] Park SW, Woo SJ, Park KH, Huh JW, Jung C, Kwon OK (2012). Iatrogenic retinal artery occlusion caused by cosmetic facial filler injections. Am J Ophthalmol.

[8] Qian H, Ling Y, Zhang M, Lenahan C, Wang C, Zheng Z, Shao A, Zhang J (2021). Massive cerebral infarction following facial injection of autologous fat: a case report and review of the literature. Front Hum Neurosci.

[9] Shen X, Li Q, Zhang H (2016). Massive cerebral infarction following facial fat injection. Aesthet Plast Surg.

[10] Szantyr A, Orski M, Marchewka I, Szuta M, Orska M, Zapala J (2017). Ocular complications following autologous fat injections into facial area: case report of a recovery from visual loss after ophthalmic artery occlusion and a review of the literature. Aesthet Plast Surg.

[11] Wang DW, Yin YM, Yao YM (2014). Internal and external carotid artery embolism following facial injection of autologous fat. Aesthet Surg J.

[12] Cuzalina A, Guerrero AV (2018). Complications in fat grafting. Atlas Oral Maxillofac Surg Clin North Am.

[13] Gornitsky J, Viezel-Mathieu A, Alnaif N, Azzi AJ, Gilardino MS (2019). A systematic review of the effectiveness and complications of fat grafting in the facial region. JPRAS Open.

[14] Ozturk CN, Li Y, Tung R, Parker L, Piliang MP, Zins JE (2013). Complications following injection of soft-tissue fillers. Aesthet Surg J.

[15] Egro FM, Coleman SR (2020). Facial fat grafting: the past, present, and future. Clin Plast Surg.

[16] Coleman SR (2020). Long-term survival of fat transplants: controlled demonstrations. Aesthet Plast Surg.

[17] Carruthers JDA, Fagien S, Rohrich RJ, Weinkle S, Carruthers A (2014). Blindness caused by cosmetic filler injection: a review of cause and therapy. Plast Reconstr Surg.

[18] Egido JA, Arroyo R, Marcos A, Jimenez-Alfaro I (1993). Middle cerebral artery embolism and unilateral visual loss after autologous fat injection into the glabellar area. Stroke.

[19] Zhou K, Cai C (2019). The successful mechanical lipectomy treatment of cerebral fat embolism following autologous fat injection. Plast Reconstr Surg Glob Open.

[20] Lee JH, Lee KH, Moon HJ (1969). A case of unilateral blindness after paraffin injection on the forehead. J Korean Ophthalmol Soc.

[21] Sotoudeh H, Shafaat O, Aboueldahab N, Vaphiades M, Sotoudeh E, Bernstock J (2019). Superior ophthalmic vein thrombosis: what radiologist and clinician must know?. Eur J Radiol Open.

[22] van der Poel NA, de Witt KD, van den Berg R, de Win MM, Mourits MP (2019). Impact of superior ophthalmic vein thrombosis: a case series and literature review. Orbit.

[23] Akhtar S (2009). Fat embolism. Anesthesiol Clin.

[24] Fukumoto LE, Fukumoto KD (2018). Fat embolism syndrome. Nurs Clin North Am.

[25] Gupta A, Reilly CS (2007). Fat embolism. Contin Educ Anaesth Crit Care Pain.

[26] Grunwald IQ, Bose A, Struffert T, Romeike BF, Politi M, Reith W, Haass A (2009). Images in neurology. Liposuction in mind. Arch Neurol.

[27] Li X, Du L, Lu JJ (2015). A novel hypothesis of visual loss secondary to cosmetic facial filler injection. Ann Plast Surg.

[28] Sito G, Manzoni V, Sommariva R (2019). Vascular complications after facial filler injection: a literature review and meta-analysis. J Clin Aesthet Dermatol.

